# The Restorative Integral Support (RIS) Model: Community-Based Integration of Trauma-Informed Approaches to Advance Equity and Resilience for Boys and Men of Color

**DOI:** 10.3390/bs13040299

**Published:** 2023-03-31

**Authors:** Stephanie Duncan, Heather Horton, Richard Smith, Bruce Purnell, Lisa Good, Heather Larkin

**Affiliations:** 1 School of Social Welfare, University at Albany, Albany, NY 12203, USA; 2 Richard Smith Speaks, Brooklyn, NY 11201, USA; 3 Love More Movement, Washington, DC 20064, USA; 4 Urban Grief, Albany, NY 12204, USA

**Keywords:** trauma, resilience, restorative practices, trauma-informed interventions, adverse childhood experiences, adverse community environments, mental health, boys and men of color, adversity, community support, social inequities, restorative integral support (RIS)

## Abstract

Mental health and health promotion research and practice have consistently revealed the social and structural inequities that boys and men of color (BMoC) face. Moreover, scholarship highlights the importance of gender, especially the concepts of masculinity and manhood, in understanding inequities that are experienced. Providers and community leaders are finding culturally relevant ways to foster healing and restoration while addressing racial trauma and the adverse community environments tied to adverse childhood experiences (ACEs). This article introduces the restorative integral support (RIS) model to promote connectivity through networks and to acknowledge the contextual differences BMoC experience when suffering from trauma and adversities. RIS is a framework used to address adversities and trauma while increasing societal awareness and advancing equity. This community-based, multidimensional approach is offered to enhance individual, agency, community, and policymaking leadership, raising awareness of mental health concerns and trauma while offering a flexible guide to developing safe spaces and support for recovery from ACEs and trauma. This article offers an in-depth appreciation of the real-life contexts within which BMoC overcome histories of adversity and trauma, demonstrating how the RIS model is applied to advance structural transformation while fostering community resilience.

## 1. Introduction

A long history of policies characterized by oppression, discrimination, and white supremacy have contributed to disproportionate poverty rates, higher incarceration rates, unemployment, and poorer health conditions among Black and brown people in the United States [1,2]. At the same time, it is difficult to identify a single period throughout United States history when white communities have not expected Black, indigenous, and people of color to overcome these obstacles, many of which are systemic and structural, by increasing their resilience [2]. In this article, boys and men of color (BMoC) are the focus of a multidimensional model that addresses the consequences of systemic racism and oppression. As with other marginalized people of color, BMoC have experienced trauma resulting from slavery, white supremacy, unsettled conflicts, and a lack of reconciliation or spaces for healing. Thus, grief from systematic loss is passed on from generation to generation as legacies, and so is the pain [3,4,5].

For hundreds of years, young Black and brown men have been stereotyped as threatening; this kind of violence extends to Black children who are assumed to be older than their actual age. The light of childhood innocence cast upon their white peers is inverted, and Black boys as young as 10 are not only more likely to be mistaken as older but are more likely to be perceived as guilty and face police violence if accused of a crime [6]. The insights, complexities, and emotions of BMoC are often portrayed in the media from a deficits-based framework [7]. However, more accurate and holistic depictions of Black people exist in movements such as the Black Joy creators [8] and the #BlackLivesMatter movement. Academics, artists, and activists have focused on joy as an ever-present but under-acknowledged force in the struggle for liberation and social justice. Part of this work entails understanding the way racism and oppression shape the lives of Black and brown people in the United States and not by merely celebrating the corporate adoption of the BLM movement [9].

This article uses a model from Integral Theory called restorative integral support (RIS) to provide a framework for recovery and healing from adversity and trauma among vulnerable populations [10]—especially those most harmed by the oppression and racism that arises through structures in our society such as the education system, court system, prisons, police, media, and industries supporting these institutions [11]. The RIS model accounts for these institutions and the broader systems of oppression influencing them (e.g., capitalism, white supremacy, patriarchy, imperialism, and settler-colonialism). Moreover, the individuality of a person makes a significant difference in traumatic stress, suggesting that post-traumatic adjustment (risk and resilience) is not solely dependent on the event itself but rather on how the event was experienced; thus, resilience might be fostered by effective intervention and policy [12]. Therefore, the application of the RIS model extends to the complicated, overlapping dynamics that influence an individual’s development across the lifespan, including inter- and intra-personal experiences. This theoretical model/approach then integrates resilience-building models (e.g., restorative justice and other restorative practices) and interventions (e.g., contextual trauma treatment model) to support those experiencing trauma and adversity within a multidimensional approach that includes a resolve to transform structures. The key elements within RIS implementation highlight the ways that leadership, policies and services, and community supports work together to facilitate resilience, recovery, and healing from adversity and trauma [10]. The RIS model is especially relevant as the U.S. is currently experiencing another “racial reckoning” [13] that has increased violence and oppression against Black and brown people and their white allies. This theory-driven article aims to expand the literature by introducing the RIS model and describing how it can be applied in service to BMoC. The multifaceted theoretical model delineated herein offers a framework for community-based leaders integrating research and indigenous knowledge to advance equity and foster community resilience. The real-world contexts within which Black and brown men overcome adversity and trauma is where the model begins. The authors offer potential next steps in which research questions can be brought forth from the community to inspire team-based research partnerships serving community knowledge development [14].

## 2. The Restorative Integral Support (RIS) Model

The RIS model was developed to support providers and community leaders in serving people experiencing multifaceted challenges in a way that emphasized strengths, resilience, and the ability to recover and heal. As a multidimensional approach to supporting the whole person, the RIS model responds to individual needs and characteristics within the context of community relationships and responsive systems. Emerging from a meta-theoretical perspective, the RIS model begins by raising awareness of both adverse childhood experiences (ACEs) and adverse community events, then by offering a flexible guide for community members to bring together readily available resources and supports (healing modalities and protective factors) while advancing policy equity and system responsiveness [10,15]. Thus, the Pair of ACEs and the need for trauma-informed, culturally relevant, and community-based interventions for BMoC is presented next. This is followed by a discussion of existing interventions that community members might want to include within the RIS framework, depending on available resources. In presenting the RIS model, the importance of strengthening positive relationships and addressing policy equity and systems transformation is emphasized as a crucial element of a truly whole person—whole community approach to maximize results. Carrying out RIS will then involve community-based research partnerships to further inform the model and its use in each community.

### 2.1. The Pair of ACEs

The original adverse childhood experiences (ACEs) research developed by Felitti, Anda, and colleagues [16] has been extended and integrated with knowledge on community adversities and historical trauma (e.g., poverty, racism, sexism, and discrimination), including the factors that influence human development across the lifespan [15,17,18,19]. Thus, in recent years, there has been a movement to acknowledge adverse community events and collectively understand trauma [20]. The Pair of ACEs was set forth by Ellis and Dietz [18] to simultaneously address adverse community environments and adverse childhood experiences, as an interconnected pair, when building community resilience. A tree depicts the Pair of ACEs—poverty, discrimination, community disruption, lack of opportunities and housing, homelessness, and violence are presented as conditions at the roots of the tree that compound adversities, including adverse childhood experiences [18]. Accumulated adverse childhood experiences—summed categories of abuse, neglect, and household challenges known as ACE scores—are powerfully correlated with costly later life health problems and have been implicated in homelessness and criminal justice involvement [10,17]. People of color may be experiencing even more damaging health effects from higher ACE scores [21].

Additionally, exposure to community violence was recently proposed as an ACE category [19]. Due to the historical and cultural context, BMoC living in poverty may be more vulnerable to high ACE scores. The Philadelphia Urban ACE Study [22] exemplifies how researchers have extended the original ACE questions to align with Black and brown men’s trauma and adversity in the U.S. Among a sample of Philadelphians asked about exposure to violence, racial/ethnic discrimination, and overall safety, Black males (ages 18–65+) reported a history of witnessing and experiencing violence at a higher rate than all racial groups, with Black adults more likely to report ACEs than white adults. The survey instruments that are inclusive, anti-racist, and generally sensitive to racial and cultural diversity are increasingly available for use [22]. An intentional focus on the most marginalized groups when developing instruments and interventions assures that all groups of people who disproportionately experience systemic oppression and disenfranchisement will not be overlooked [23].

In the wake of George Floyd’s murder and increasing discussions about racial trauma, that is, the individual and collective psychological distress that results from experiencing or witnessing discrimination, threats of harm, violence, and intimidation directed at ethno-racial minority groups, there is a need to integrate approaches to healing that use wisdom derived from Black, brown, and indigenous communities [24], (p. 53). Fusing ACE and trauma-informed principles into community-based approaches allows access to more culturally responsive services and support and enables equitable policies to respond to mental health disparities among BMoC [21]. The prevalence of trauma exposure among BMoC has created a push for programming to facilitate culturally appropriate healing for the population. Applying the RIS model involves integrating evidence-supported interventions and indigenous practices while building on community strengths. This approach addresses the various forms of trauma and adversity experienced within communities while simultaneously supporting community leaders as activists to advance equitable policies and systems responsive to the Pair of ACEs in fostering resilience [10,25].

### 2.2. Importance of Trauma-Informed, Culturally Sensitive Community-Based Interventions

Trauma results from “an event, series of events, or set of circumstances that is experienced by an individual as physically or emotionally harmful or life-threatening and that has lasting adverse effects on the individual’s functioning and mental, physical, social, emotional, or spiritual well-being” [26]. Accumulated early adversities can cascade into health and social problems throughout one’s lifespan [27]. According to Philpart and Bell [28], to support the growth, development, and capacity with which to realize opportunities for BMoC, strategies that include spiritual healing and culturally relevant trauma-informed practices are needed to improve and enhance outcomes. A trauma-informed approach “includes trauma-specific interventions, whether assessment, treatment or recovery supports, yet it also incorporates key trauma principles into the organizational culture” [26].

Occurrences exacerbating trauma experienced by BMoC can be compounded when seeking support from mental health providers that lack an anti-racism consciousness. Educators and mental health workers do not always understand the cultural and racial support that is most beneficial for men of color [29]. While there are many evidence-supported interventions, protocols, and programs designed to support those with a history of adversity and trauma, there is an identified need to improve how we address issues of culture and context in mental health services [30]. The mental health field is influenced by practices rooted in a culture of white supremacy. Systems embedded with white supremacist ideologies dehumanize non-white racial groups and negate their perspectives and lived experiences [31]. Studies that directly identify the trauma and adversity experienced by BMoC are on the rise, based on extensive evidence that African Americans are more likely to be involuntarily committed, placed in solitary or restraints, and treated with higher doses of psychotropic medication than their white counterparts [32,33]. Cusack and colleagues [33] and, more recently, DeAngelis [34] have suggested that boys and men of color receive less comprehensive care, have less access to experienced providers, and are faced with fewer resources, while at the same time their mental health is challenged by racism. There continues to be a notable disparity between the prevalence of schizophrenia in people of color in the United States and Canada and their white counterparts in these same countries, with people of color receiving a higher diagnosis rate [35]. The overdiagnosis of Black and brown people is deeply rooted in the racial bias of practitioners and further illustrates the argument that service providers have not been trained to acknowledge or treat the trauma present within this group.

The inequities experienced by BMoC across sectors highlight the extent of the ongoing work required, particularly in dismantling systems that harm the most vulnerable members of our society e.g., [36]. For these reasons, cultural humility is increasingly an expected part of mental health practice, as service providers and systems-oriented strategies are emerging to express and address the specific needs of BMoC [37]. Reflexive work strengthens cultural humility and includes focusing on knowledge of self, accurately perceiving cultural values, recognizing the dynamic nature of culture, and challenging systemic barriers faced by marginalized individuals and communities [2]. Nonetheless, while social workers and other service providers seek to promote social and racial justice, a comprehensive and nuanced understanding of social inequities is often lacking. There is a need to develop curricula for mental health practitioners that nurture an anti-racist consciousness [38] via trauma-informed perspectives.

### 2.3. Positive Relationships, Policy Equity, and Resilience

Adverse childhood experiences, community violence, racism, and other social inequities place BMoC at risk of exposure to contextual factors that can harm their psychological well-being. While the causes of violence are complex, salient factors are associated with community violence in urban neighborhoods. Over-policing and harsh criminal policies are other oppressive forces that tend to break the community’s trust in police departments and, generally, in the criminal justice system. Simultaneously, the same disadvantaged communities receive inferior service and protection from the police [39]. BMoC experience relational disruptions caused by racial trauma, violence, incarceration, and health-related deaths, further presenting challenges to the construction of positive relationships necessary to increase resilience [40]. BMoC communities have been significantly fragmented and disproportionately affected by various forms of trauma and adversity in the United States.

It is important to note that, as part of improving self-regulation, trauma survivors benefit from positive relationships that may not have been present during childhood. Positive relationships aid in recovery from the trauma of violence and victimization, and trust, reconnection, and safety are essential elements of healing [41,42]. In part, healing is thus contingent upon restoring broken connections with one’s cultural identity [27]. Building positive relationships can help BMoC feel safe and trust others, learn new ways to relate to people, and develop compassion [42,43]. A recent study found that strong familial relationships, parental bonding, and a positive school climate can help Black youths build resilience in the face of exposure to community violence [44]. Furthermore, leveraging relationships with leaders in the community, such as teachers, barbers, or persons of faith, can help destigmatize conversations about mental health [29].

A unique and growing strategy that appreciates the value of existing community relationships is barbershop-based interventions. This approach recognizes barbers as change agents and individuals who promote resilience in the community [45]. Hill et al. [45] responded to the importance of engaging partnerships by valuing community members as equal collaborators, thereby enhancing research on interventions more suitable for this group. Restorative practices represent another positive intervention strategy, rooted in relationships and repairing harm through conflict resolution. Moreover, restorative practices foster resilience and communication across the individual, interpersonal, and collective realms, successfully illustrating empathy and improving relationships [46]. Focusing on the community as a whole rather than changes in individual behavior produces a more significant impact [47]. For example, Woods-Jaeger et al. [48] conducted a study using the Radical Healing Framework, a strengths-based model that activates resilience to oppression to foster wellness across individual, interpersonal, and community dimensions. The participating youth believed they could function well and enhance resilience after exposure to violence by engaging in community activities of dance, art, music, and sports [48].

Lanni [39] proposes that communities develop restorative justice programs as an alternative to the traditional criminal process for some crimes. Many communities have begun experimenting with programs to reduce the role of police (CAHOOTS program in Eugene, OR; The Cure Violence in Chicago, IL). Programs such as these are alternatives to the traditional criminal process and not only reduce the effects of harsh punishments on marginalized communities but also promote community safety [39]. Additionally, restorative justice has implications for truth and reconciliation that repair the costs of ruptured relationships resulting from white supremacy. Perhaps racial justice could be advanced by creating a U.S. Truth and Reconciliation Commission that would attend to the historical trauma connected to slavery and racism [49]. For example, reparations could involve community investments to break the trajectories of the Pair of ACEs and historical trauma by advancing policy equity and transforming structures while fostering resilient communities and bringing together evidence-supported and indigenous healing modalities within communities. Recognizing the high costs of ACEs, investments in preventing ACEs and their consequences are expected to have significant payoffs for society as a whole [10,17].

Thus, in light of the Pair of ACEs and increased attention to understanding trauma collectively [20], attention to nurturing positive relationships and community experiences are essential goals when working with BMoC. The experiences of community violence and other social inequities BMoC encounter necessitate a tailored and inclusive approach to promoting resilience. To better understand resilience among Black and brown people, it is essential to understand these individuals’ lived experiences and perspectives in all spheres, i.e., individual, interpersonal, and institutional [48]. Furthermore, grassroots activism is called for to address trauma consequences, advocate for appropriate responses by policymakers and stakeholders, and forge cross-sector collaborations to achieve these goals [25]. By examining the interconnected factors contributing to adversity and trauma (i.e., trauma related to assault and abuse, violence, or racism), providers, agencies, and community leaders will be better prepared to provide compassionate support, including meaningful interventions and culturally relevant leadership opportunities for Black and brown boys and men [50]. Furthermore, since relational ruptures take place within the context of structural racism, this warrants the transformation of policies and systems to advance equity alongside the development of relationships and protective factors to foster resilience [18]. Service providers and community leaders have essential roles in raising awareness about the human and societal costs of adverse childhood experiences (ACEs), adverse community events (including racism and oppression), and other social determinants of health. Community and team-based research partnerships are needed to build upon knowledge of what works well for BMoC within community relationships while exploring how nurturing communities are strengthened as policies and systems are transformed and equity is advanced.

### 2.4. A Multidimensional Framework for RISing Out of Trauma

**Figure 1:**
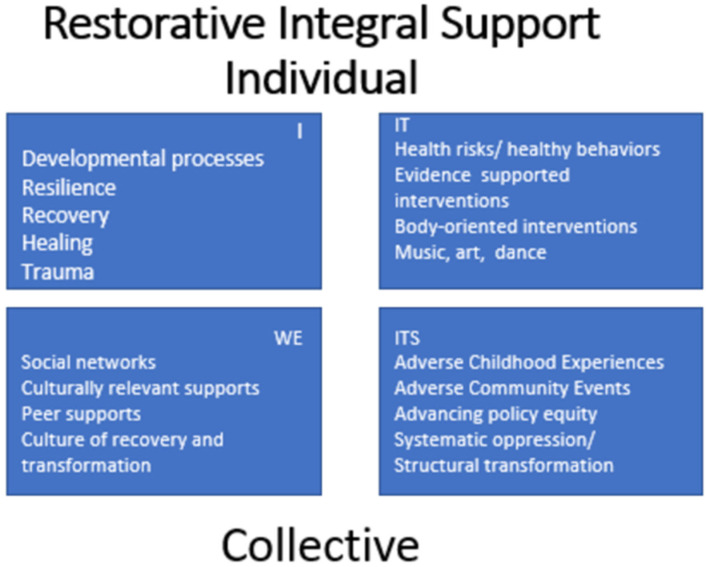
A quadrant perspective on ACEs and resilience.

ACEs, including exposure to community violence, other social determinants of health, and health risk behaviors, can interfere with one’s ability to resolve one stage of development and move on to the next [10,17]. When applying RIS, development is recognized as involving “all quadrants” (Figure 1). For example, individual interior cognitive and socio-emotional developmental processes (I quadrant) take place in the context of community relationships that involve shared values and meaning-making (WE quadrant), as well as policy, systemic, and structural environmental influences (ITS quadrant), all of which arise along with an individual’s observable coping behaviors (IT quadrant). The quadrant labels of “I”, “WE”, “IT”, and “ITS” are short-hand denotations for the first-person (“I”), second-person (“WE”), third-person singular (“IT”), and third-person plural (“ITS”) perspectives. Noting that all cultural and linguistic groups recognize first-, second-, and third-person perspectives, integral theory contends that embracing all of these dimensions of experience is at the heart of being fully inclusive [51]. The right-hand quadrants (IT and ITS) represent observable aspects of individuals and systems. The left-hand quadrants (I and WE) are subjective and involve asking questions to discover individual and community values and to create meaning. The four quadrants evolve together, representing different dimensions of experience [10,15,52]. Perceiving how the four quadrants arise together and reflect one another facilitates a consideration of the varied aspects of the “whole person—whole community” for a multifaceted approach (see Figure 1).

ACEs are observable events within systemic interactions, and the families and communities within which ACEs occur often experience institutionalized and systemic discrimination. These observable systemic interactions map onto the “ITS” quadrant. Structural racism and other historical and current policies that shape or limit service delivery and access are also mapped to the “ITS” quadrant. Self-care behaviors, such as building resilience through dance and art therapy and individually oriented evidence-supported interventions, are mapped to the “IT” quadrant. Health risk behaviors (which ACE authors often point out are attempted behavioral solutions or coping strategies), neurodevelopment, and health are also mapped to the “IT” quadrant. Positive relationships, peer supports, grassroots activism, restorative justice, and other community-oriented restorative and resilience-building practices are mapped to the “WE” quadrant. The culture of white supremacy and racism, along with the social taboos that keep ACEs and other traumatic experiences hidden, are also mapped to the “WE” quadrant. Interior developmental processes, emotions, individual capacities for resilience, life skills, and the experience of trauma are mapped to the “I” quadrant. (See Figure 1).

The RIS model highlights that adversities co-arise with various inner and outer resources, with trauma emerging as an “all-quadrant” experience. As described, adverse events are typically observable within systemic interactions (ITS quadrant); moreover, whether or not any event has the potential to derail developmental processes or is subjectively experienced as overwhelming or traumatic (I quadrant) has a lot to do with what other resources and supports versus stressors are taking place in all quadrants. The RIS model also emphasizes that the ongoing systematic oppression of Black and brown people (ITS quadrant) must be included and addressed within a multidimensional intervention approach, advancing policy equity (ITS quadrant) along with culturally relevant supports (WE quadrant) to facilitate individual resilience, recovery, and healing (I quadrant) and healthy behaviors (IT quadrant) that work together to strengthen communities. Without transforming racist structures or advancing policy equity, piecemeal approaches to addressing the Pair of ACEs and trauma will likely fall short in effectiveness.

As service providers begin to conceptualize the timeline of historical trauma for BMoC, they will understand how psychological and emotional consequences of mass traumatic experiences can be spread over time to ensuing generations through physiological, environmental, and social pathways [53]. Thus, changing these pathways has been connected to spirituality, hope, and the culture of the BMoC community throughout a history that has never been void of systemic oppression, racism, and trauma [54]. The all-quadrants approach can also be used to uncover the ways that trauma is intergenerational. For example, adults who have experienced ACEs (ITS quadrant) and trauma (I quadrant) in childhood may adopt health risk behaviors (IT quadrant) that later become ACEs (ITS quadrant) for their children, with the potential for these children to experience trauma (I quadrant). These outcomes may have much to do with the availability of positive relationships and community resources (WE quadrant). It can be helpful to picture feedback loops among the quadrants [10]. In this way, the RIS model may be useful for a multifaceted approach to the whole person–whole community healing from intergenerational trauma for BMoC. While guiding us to touch base with all quadrant perspectives in addressing adversity and trauma, the framework allows flexibility in choosing the most practical intervention opportunities within BMoC communities [10]. It is then expected that the next steps in research and knowledge development will further inform what RISing out of trauma looks like for BMoC in various resilient communities [14,55].

This multi-perspectival awareness expands opportunities to intervene to both prevent and address adverse events or trauma. A vital aspect of the RIS model is to enhance agency, community, and policymaking leadership by raising awareness of ACEs, trauma, resilience, and the contexts within which they occur. Community leaders set an example through behaviors, role modeling, and relationship-building, setting the tone for the culture around them. Furthermore, RISing leaders work together to change policies and procedures to facilitate healthy programs and community contexts. There is recognition that we all contribute to the cultures of our programs and communities. We can also work together to transform and recreate policies, systems, and structures to facilitate well-being and through which joy can be shared [10]. With the multidimensional, all-quadrant approach of the RIS model, we expect that the next steps in community research partnerships will include tracking individual and community resilience outcomes and an exploration of the processes and pathways to resilience, well-being, and joy in Black and brown communities, thereby further building knowledge of the many strengths and resources within Black and brown communities. It is anticipated that ongoing team-based research will demonstrate the power of this multidimensional anti-racist approach and the power of Black and brown people and their communities, further informing the RIS model and its implementation more broadly, see also [14,55].

### 2.5. Mapping an Example to the Quadrants

Historically, barbershops have been a haven for Black boys and men of color. As Black-owned and Black-patronized barbershops emerged in the early 20th century, the barbershop in the African American community was also where Black men could gather without inhibition and worry [56]. It was and remains an essential space for connection and community. Critical issues are discussed that directly impact their lives: racial profiling, the shootings of unarmed Black men by police, and racial discrimination in the workplace [57]. Help-seeking frequently occurs in informal gatherings as a way for BMoC to seek mental health services that are often stigmatized by peers. The Confess Project [58], founded in 2016 by Lorenzo Lewis, is a coalition to create a safe space for Black and brown boys to discuss mental health issues. Lorenzo was born in jail to an incarcerated mother (WE quadrant, i.e., family and community relationships; ITS quadrant, i.e., criminal justice system and its influence on the family system). He struggled with depression, anxiety, and anger throughout his youth (I quadrant, i.e., overwhelming emotions). He broke through the prison cycle and committed to addressing his mental health concerns (IT quadrant, i.e., self-care behaviors). Lewis became a mental health advocate and spoke at churches, schools, and conferences, sharing his story (IT quadrant, i.e., leadership behavior/speaker; WE quadrant, i.e., community activism). He later decided to create a peer support model for barbers to become mental health advocates and create a safe space for boys and men of color to discuss mental health (WE quadrant, i.e., peer support). He knew the importance and impact that beauty and barbershops could make given their presence among BMoC (IT quadrant, i.e., what others observe of the individual). Lewis has created the Beyond the Barber Shop program, training barbers to become mental health advocates, and has successfully engaged 600 barbers in 35 cities and 14 states (WE quadrant, i.e., community champions, social networks). Large organizations such as Toyota, Andi’s, and Gillette have helped fund this national grassroots organization, and the Confess Project [58] participated in a Harvard study that confirmed the vital role of Black barbers as change agents for positive mental health outcomes (I and IT quadrants) in Black communities (WE quadrant, i.e., community coalition; ITS quadrant, i.e., systems- barbershops). Barbers and other community leaders serve as change agents and set examples through behaviors and role-modeling (IT quadrant), setting a positive tone for the culture surrounding them and leading to healthier communities (WE quadrant). In this way, the Confess Project [58] has been applied to the four quadrants, noting the multiple dimensions (individual/collective and interior/exterior influences) evident in the development of the program. The project demonstrates the usefulness of the multidimensional framework offered by the RIS model. Moreover, it reflects how an all-quadrant perspective can enhance individual agency, community, and policymaking leadership by expanding opportunities for intervention from different perspectives.

## 3. Conclusions and Discussion

This article focused on applying a theoretical model called restorative integral support to the real-world experiences of racism and ACEs for boys and men of color. While the current article focused on BMoC and their adverse experiences, it is also essential to consider other aspects of their identity, such as sexual orientation, socioeconomic status, or country of origin. Furthermore, it is imperative to recognize that girls and women of color also have unique needs and face adversities and challenges, pointing to key next steps in the application of the model. The inequities BMoC face are considered in the context of a lack of trauma-focused treatment modalities that support this group. People of color have long demonstrated resilience by garnering support from outside traditional mental health settings because they have not been proven to foster resilience and support. Additionally, studies identifying trauma and adversity experienced by BMoC when receiving services are minimal. While evidence-supported interventions exist, including protocols designed to support people with a history of adversity and trauma, there is an identified need to improve how we address issues of culture and context in mental health services [30,59]. 

Applying the RIS model to BMoC guides the examination and re-shaping of systems, procedures, and policies (ITS quadrant) while identifying ways to intentionally develop restorative and healing cultural contexts (WE quadrant) within which individually oriented interventions building on strengths (I quadrant) and supporting healthy behaviors (IT quadrant) are offered. For example, this might involve (1) developing evidence-supported interventions (IT quadrant) that emphasize BMoC’s strengths (I quadrant), (2) increasing access to services and redesigning systems to improve services (ITS quadrant), (3) engaging in grassroots activism (WE quadrant) to address systemic injustices through policy advocacy (ITS quadrant), (4) creating healing spaces and addressing intergenerational racism and trauma (WE quadrant), and (5) supporting indigenous neighborhood leaders who offer role-modeling (IT quadrant), relationship building, and peer supports (WE quadrant). Acknowledging the need for unique interventions to work with each population or person, RIS allows flexibility in building upon the best local practices. Some practice examples that can be included in the RIS model are barbershop and church-based intervention models, restorative practices and restorative justice, trauma resilience, and contextual trauma therapy. An outreach devoted to Black men’s mental health and well-being suggests the importance of engagement in the context of race, culture, and gender [60]. Incorporating appropriate ways to foster BMoC’s mental health needs will include addressing racial trauma and its effects beyond adapting individual therapy, calling for a truly multidimensional approach to racially just trauma-informed supports.

The RIS model has the potential to facilitate relationships between BMoC, mental health practitioners and/or community leaders, and prevention scientists to create a space in which BMoC are recognized as experts in their lived experiences and who can identify multidimensional outcomes (i.e., individual, interpersonal relationships and social networks, community and institutional change in policies, practice, and procedures) that can be studied through evidence-based research e.g., [61]. Key training implications for supporting trauma health promotion are: (1) Learn from and support leaders focused on the needs of BMoC and other minoritized groups, offering RIS guidance and assistance in bringing together approaches chosen by the community, which could include indigenous healing, restorative practices, barbershop-based interventions, and policy advocacy (2) Support the development of leaders who intentionally cultivate healthy social networks, such as those promoting culturally sensitive mental health and peer supports as well as grassroots activists promoting policy equity and systems redesign, recognizing that these are all aspects of a multidimensional intervention that includes the intentional development of community contexts.

BMoC’s mental health is framed by several layers of gendered racism that affect their individual and interpersonal experiences and institutional opportunities [62]. The RIS model is presented to address this gap by including both trauma-informed practice and societal change. The goal is to recognize and support the flourishing of Black and brown communities, which continues even in the face of incremental change in challenging contexts. The RIS model is offered as a resource for agencies, community members, and educational institutions when explicitly focusing on BMoC who have experienced trauma and adversity by fostering resilience while advancing policy equity, including transforming the structures through which services are often provided. RIS integrates research and advances multifaceted community-based team research to build knowledge relevant to the communities served. This article aims to show the usefulness of a theory-based model in real-world community settings that facilitates community partnerships for team-based research. The RIS model is proposed as a useful “whole person—whole community” approach with the potential to expedite transformational change in society while advancing pathways for healing within communities and research partnerships for knowledge development that further informs the model.

## Data Availability

This is a theoretical article and there was no research data collected.
